# Supplementary material for: A Humanized Yeast Phenomic Model of Deoxycytidine Kinase to Predict Genetic Buffering of Nucleoside Analog Cytotoxicity
Source: Genes (Basel). 2019 Sep 30;10(10):770. doi: 10.3390/genes10100770 (PMC6826991; doi:10.3390/genes10100770)
Supplement: Supplementary file 1 [file genes-10-00770-s001.zip › Additional_Files/Additional_File7_GOTermHeatmaps.zip!/Additional_File7_GOTermHeatmaps/transcription_factor_complex.pdf]

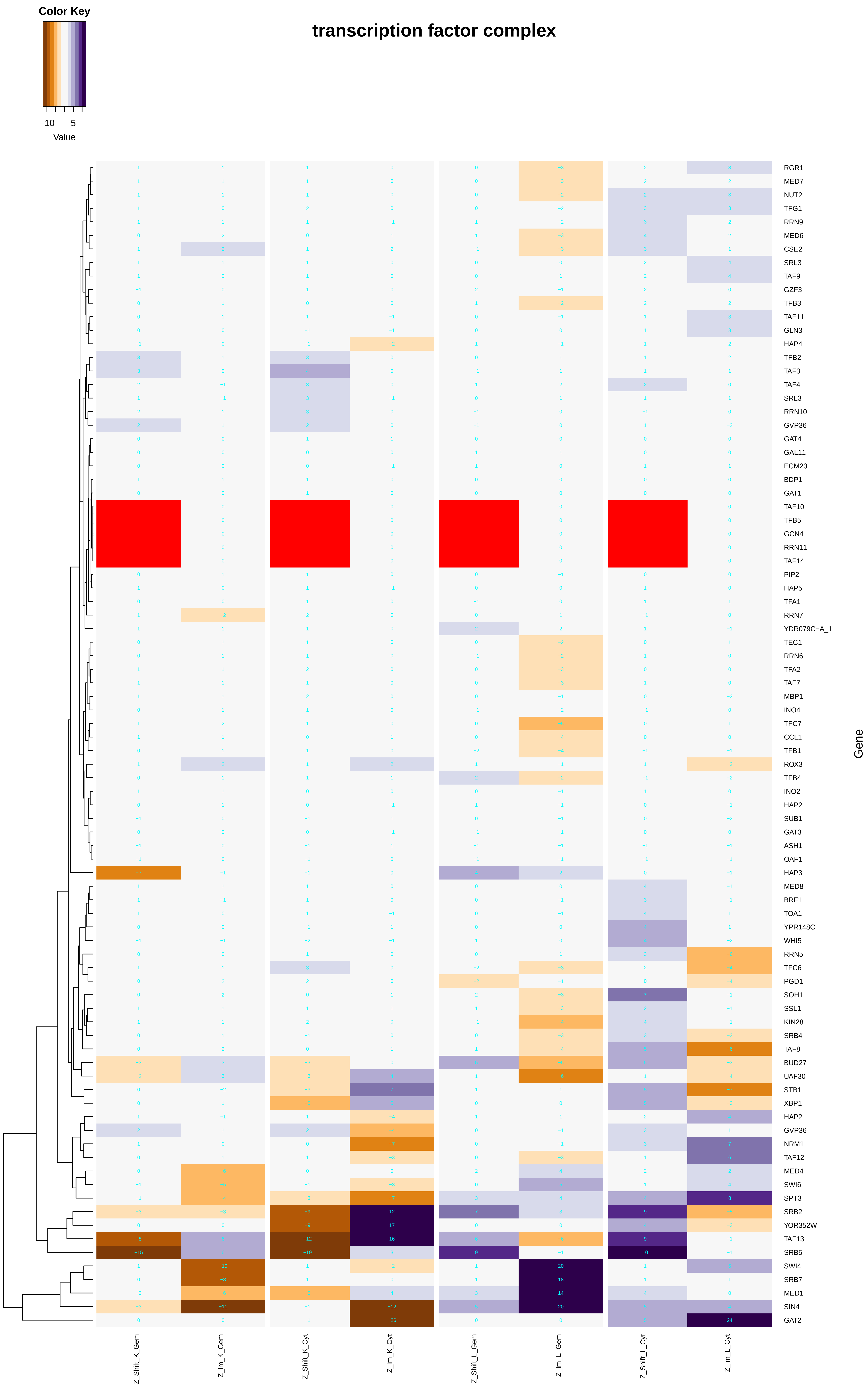
transcription factor complex
Color Key
−10
5
Value
Gene
RGR1
MED7
NUT2
TFG1
RRN9
MED6
CSE2
SRL3
TAF9
GZF3
TFB3
TAF11
GLN3
HAP4
TFB2
TAF3
TAF4
SRL3
RRN10
GVP36
GAT4
GAL11
ECM23
BDP1
GAT1
TAF10
TFB5
GCN4
RRN11
TAF14
PIP2
HAP5
TFA1
RRN7
YDR079C−A_1
TEC1
RRN6
TFA2
TAF7
MBP1
INO4
TFC7
CCL1
TFB1
ROX3
TFB4
INO2
HAP2
SUB1
GAT3
ASH1
OAF1
HAP3
MED8
BRF1
TOA1
YPR148C
WHI5
RRN5
TFC6
PGD1
SOH1
SSL1
KIN28
SRB4
TAF8
BUD27
UAF30
STB1
XBP1
HAP2
GVP36
NRM1
TAF12
MED4
SWI6
SPT3
SRB2
YOR352W
TAF13
SRB5
SWI4
SRB7
MED1
SIN4
GAT2
Z_Shift_K_Gem
Z_Im_K_Gem
Z_Shift_K_Cyt
Z_Im_K_Cyt
Z_Shift_L_Gem
Z_Im_L_Gem
Z_Shift_L_Cyt
Z_Im_L_Cyt

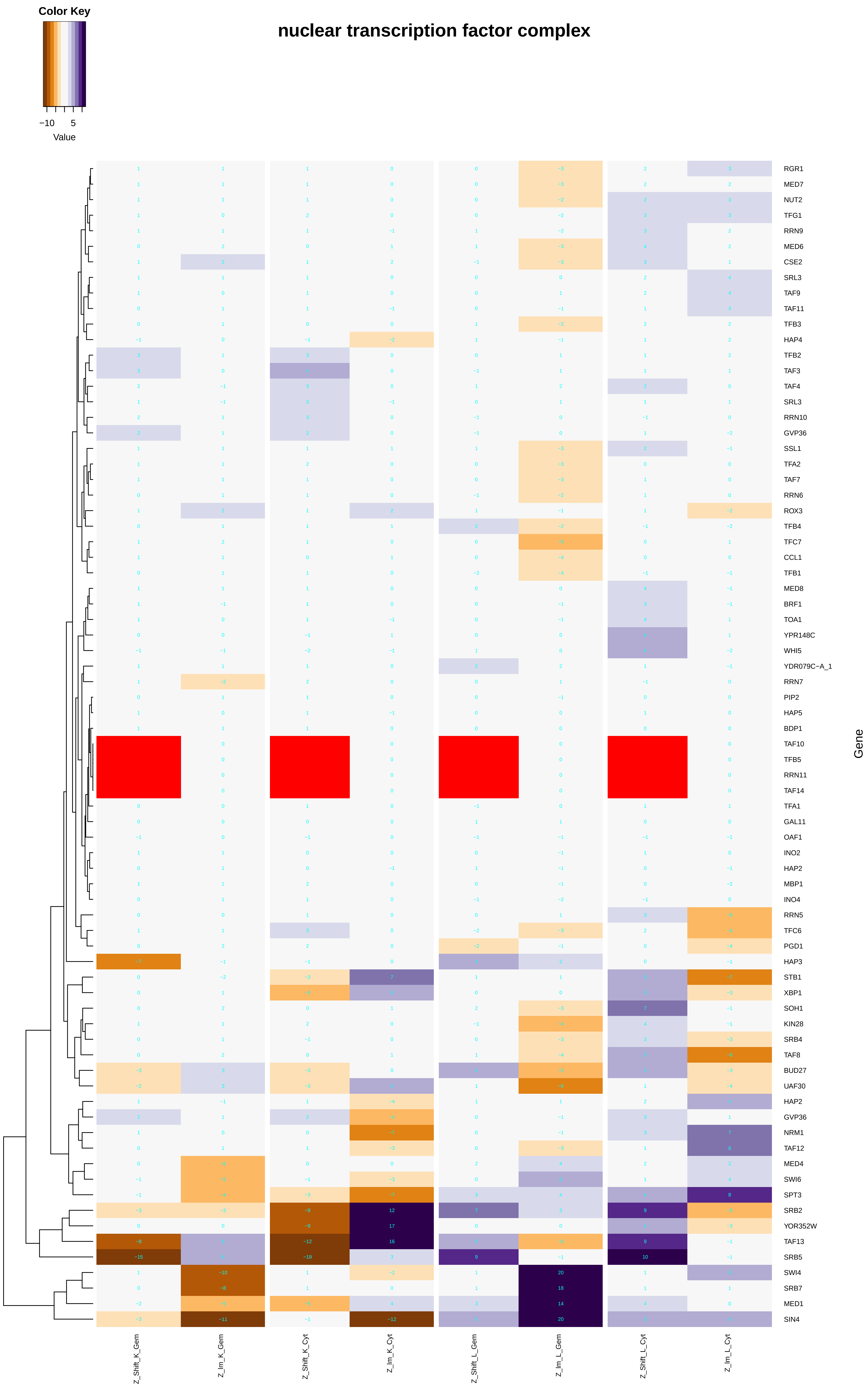
nuclear transcription factor complex
Color Key
−10
5
Value
RGR1
MED7
NUT2
TFG1
RRN9
MED6
CSE2
SRL3
TAF9
TAF11
TFB3
HAP4
TFB2
TAF3
TAF4
SRL3
RRN10
GVP36
SSL1
TFA2
TAF7
RRN6
ROX3
TFB4
TFC7
CCL1
TFB1
MED8
BRF1
TOA1
YPR148C
WHI5
YDR079C−A_1
RRN7
PIP2
HAP5
BDP1
TAF10
TFB5
RRN11
TAF14
TFA1
GAL11
OAF1
INO2
HAP2
MBP1
INO4
RRN5
TFC6
PGD1
HAP3
STB1
XBP1
SOH1
KIN28
SRB4
TAF8
BUD27
UAF30
HAP2
GVP36
NRM1
TAF12
MED4
SWI6
SPT3
SRB2
YOR352W
TAF13
SRB5
SWI4
SRB7
MED1
SIN4
Gene
Z_Shift_K_Gem
Z_Im_K_Gem
Z_Shift_K_Cyt
Z_Im_K_Cyt
Z_Shift_L_Gem
Z_Im_L_Gem
Z_Shift_L_Cyt
Z_Im_L_Cyt

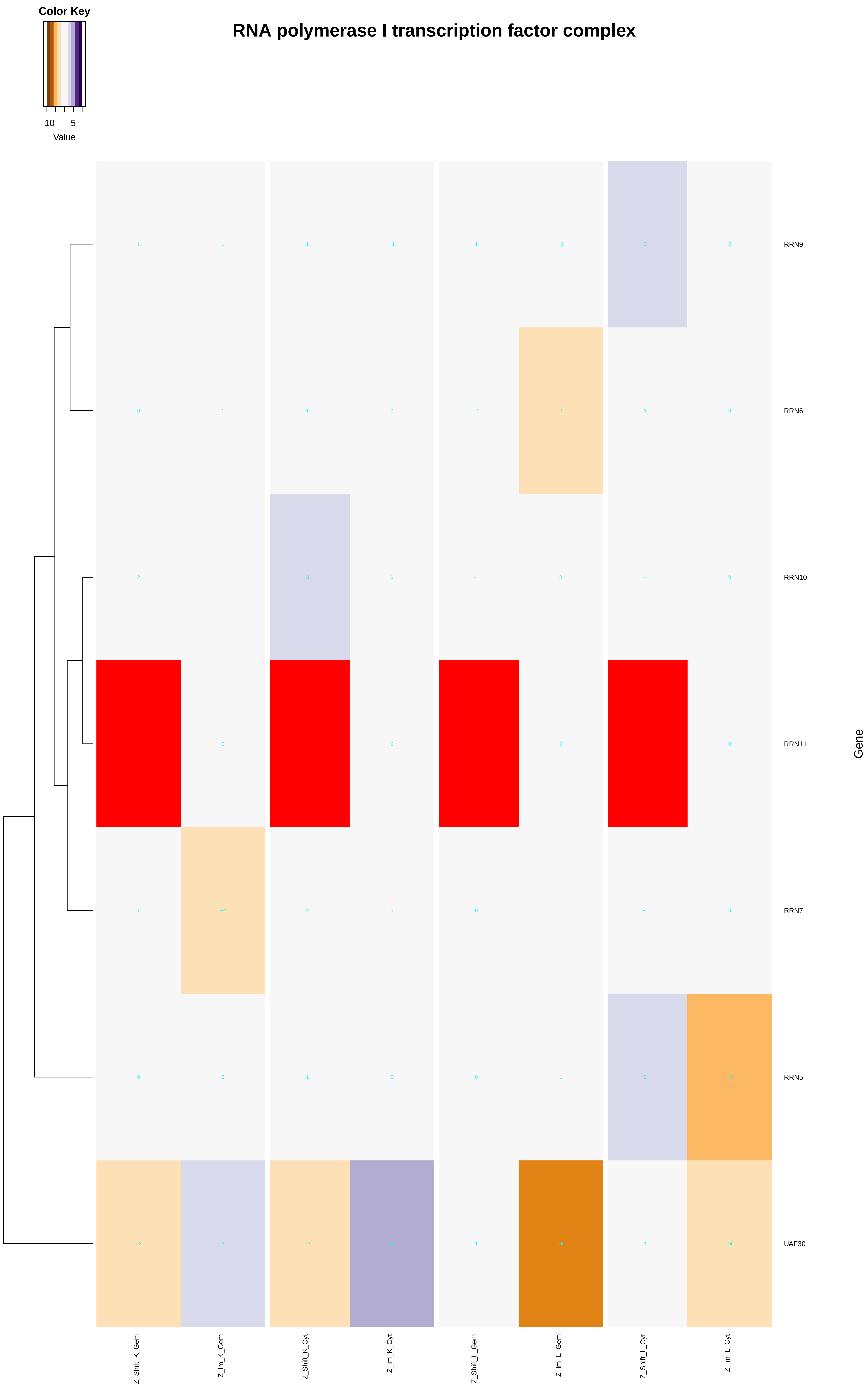
RNA polymerase I transcription factor complex
Color Key
−10
5
Value
RRN9
RRN6
RRN10
RRN11
RRN7
RRN5
UAF30
Gene
Z_Shift_K_Gem
Z_Im_K_Gem
Z_Shift_K_Cyt
Z_Im_K_Cyt
Z_Shift_L_Gem
Z_Im_L_Gem
Z_Shift_L_Cyt
Z_Im_L_Cyt

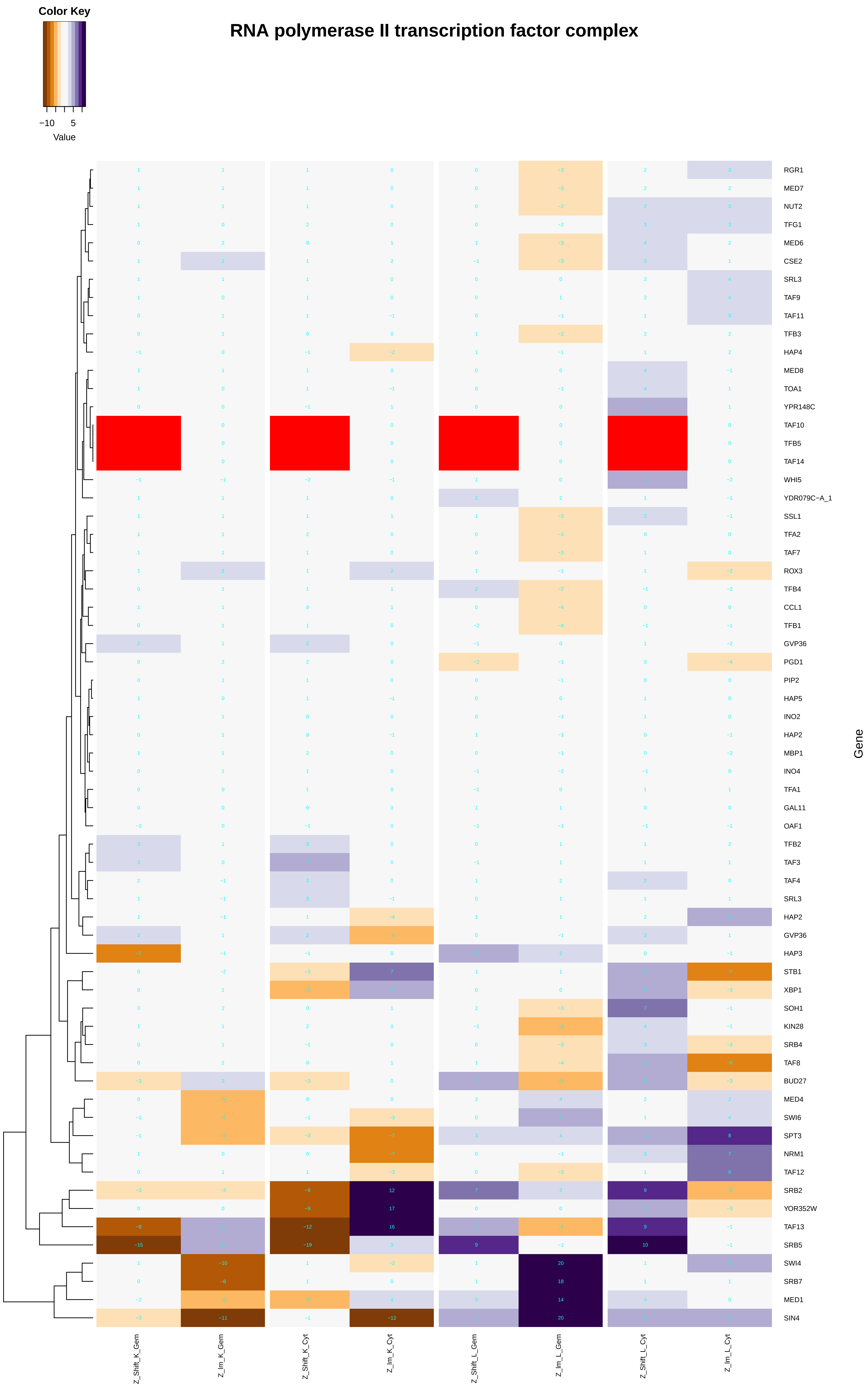
RNA polymerase II transcription factor complex
Color Key
−10
5
Value
RGR1
MED7
NUT2
TFG1
MED6
CSE2
SRL3
TAF9
TAF11
TFB3
HAP4
MED8
TOA1
YPR148C
TAF10
TFB5
TAF14
WHI5
YDR079C−A_1
SSL1
TFA2
TAF7
ROX3
TFB4
CCL1
TFB1
GVP36
PGD1
PIP2
HAP5
INO2
HAP2
MBP1
INO4
TFA1
GAL11
OAF1
TFB2
TAF3
TAF4
SRL3
HAP2
GVP36
HAP3
STB1
XBP1
SOH1
KIN28
SRB4
TAF8
BUD27
MED4
SWI6
SPT3
NRM1
TAF12
SRB2
YOR352W
TAF13
SRB5
SWI4
SRB7
MED1
SIN4
Gene
Z_Shift_K_Gem
Z_Im_K_Gem
Z_Shift_K_Cyt
Z_Im_K_Cyt
Z_Shift_L_Gem
Z_Im_L_Gem
Z_Shift_L_Cyt
Z_Im_L_Cyt

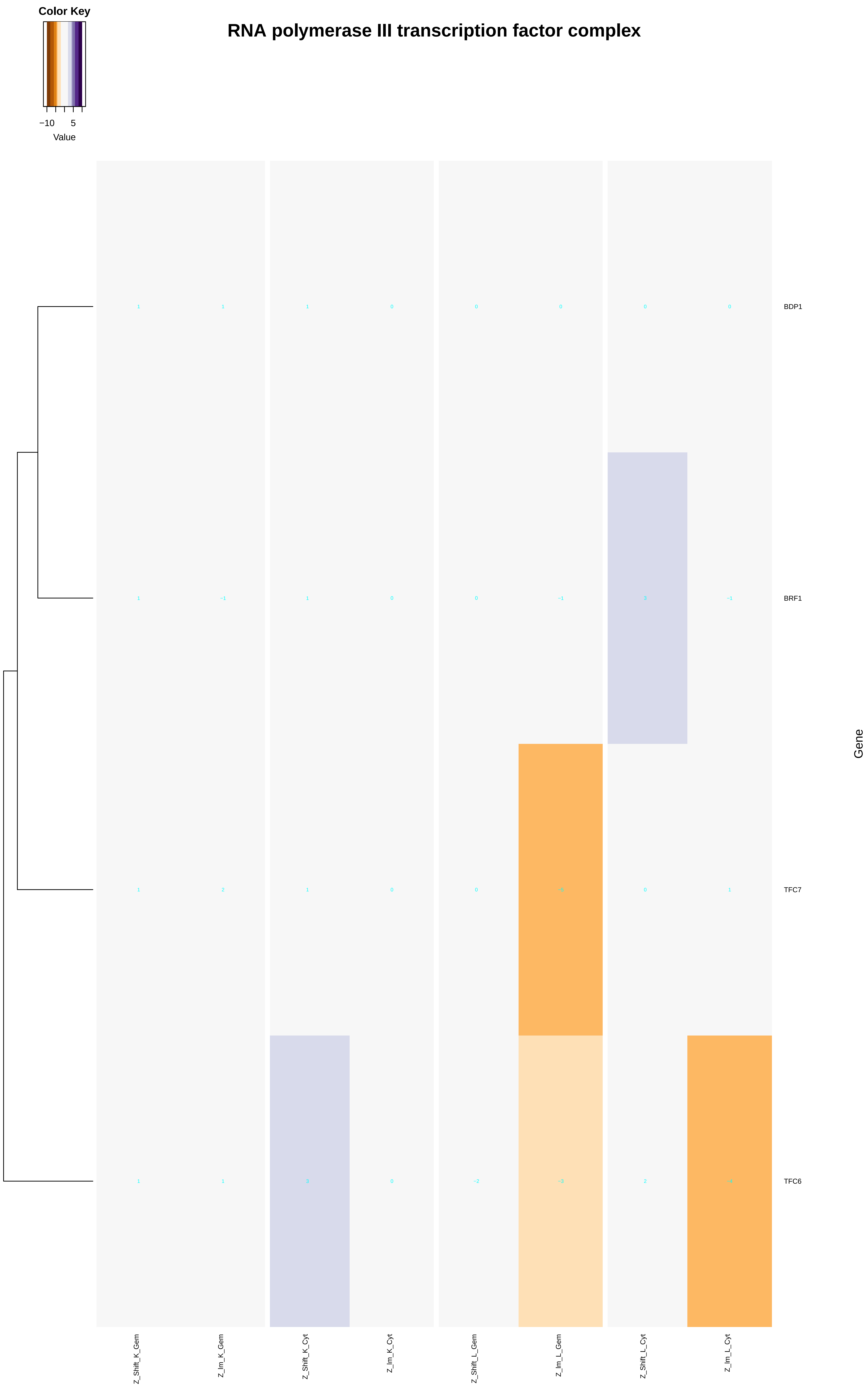
RNA polymerase III transcription factor complex
Color Key
−10
5
Value
BDP1
BRF1
TFC7
TFC6
Gene
Z_Shift_K_Gem
Z_Im_K_Gem
Z_Shift_K_Cyt
Z_Im_K_Cyt
Z_Shift_L_Gem
Z_Im_L_Gem
Z_Shift_L_Cyt
Z_Im_L_Cyt

RNA polymerase I upstream activating factor complex

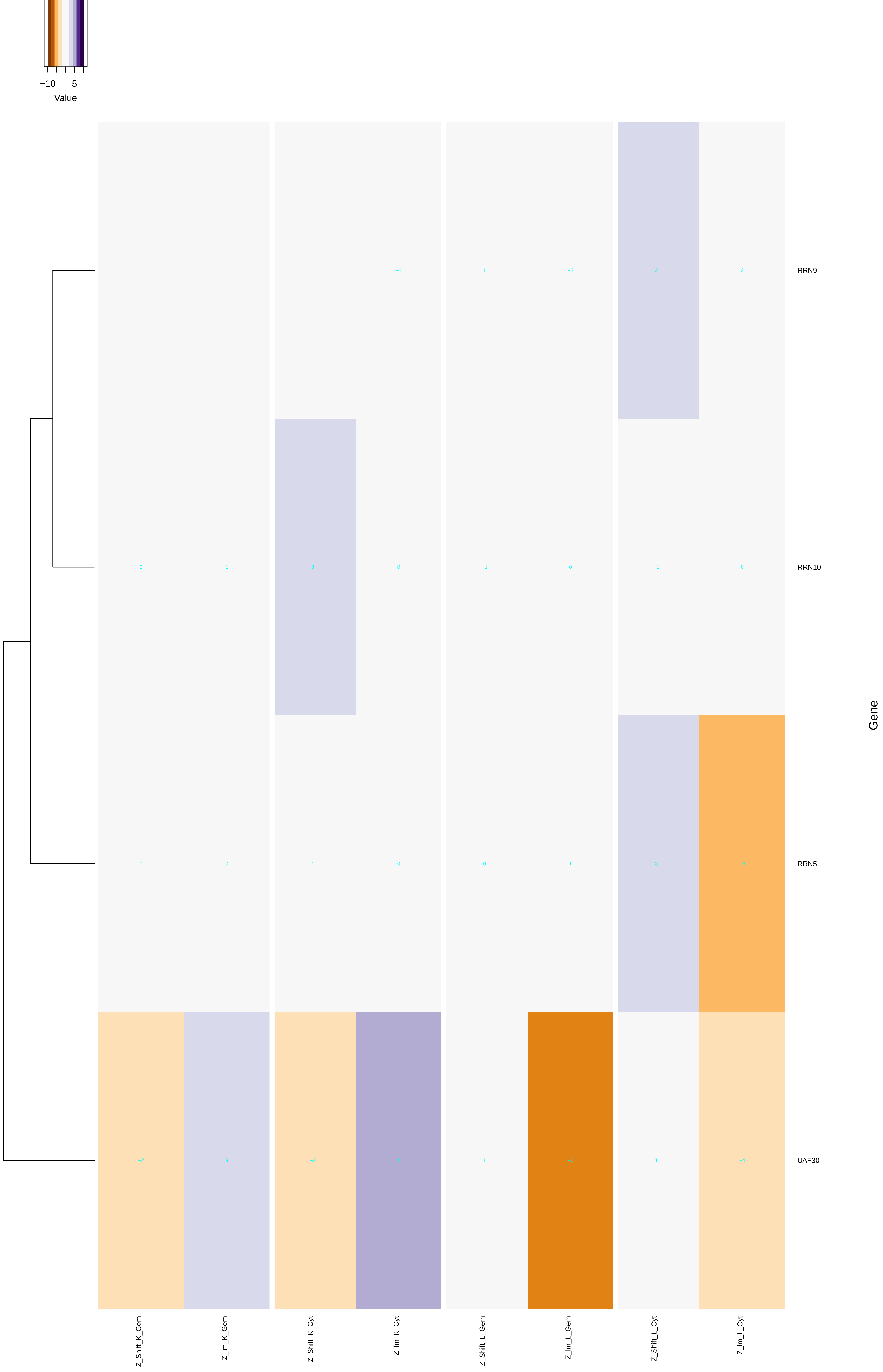

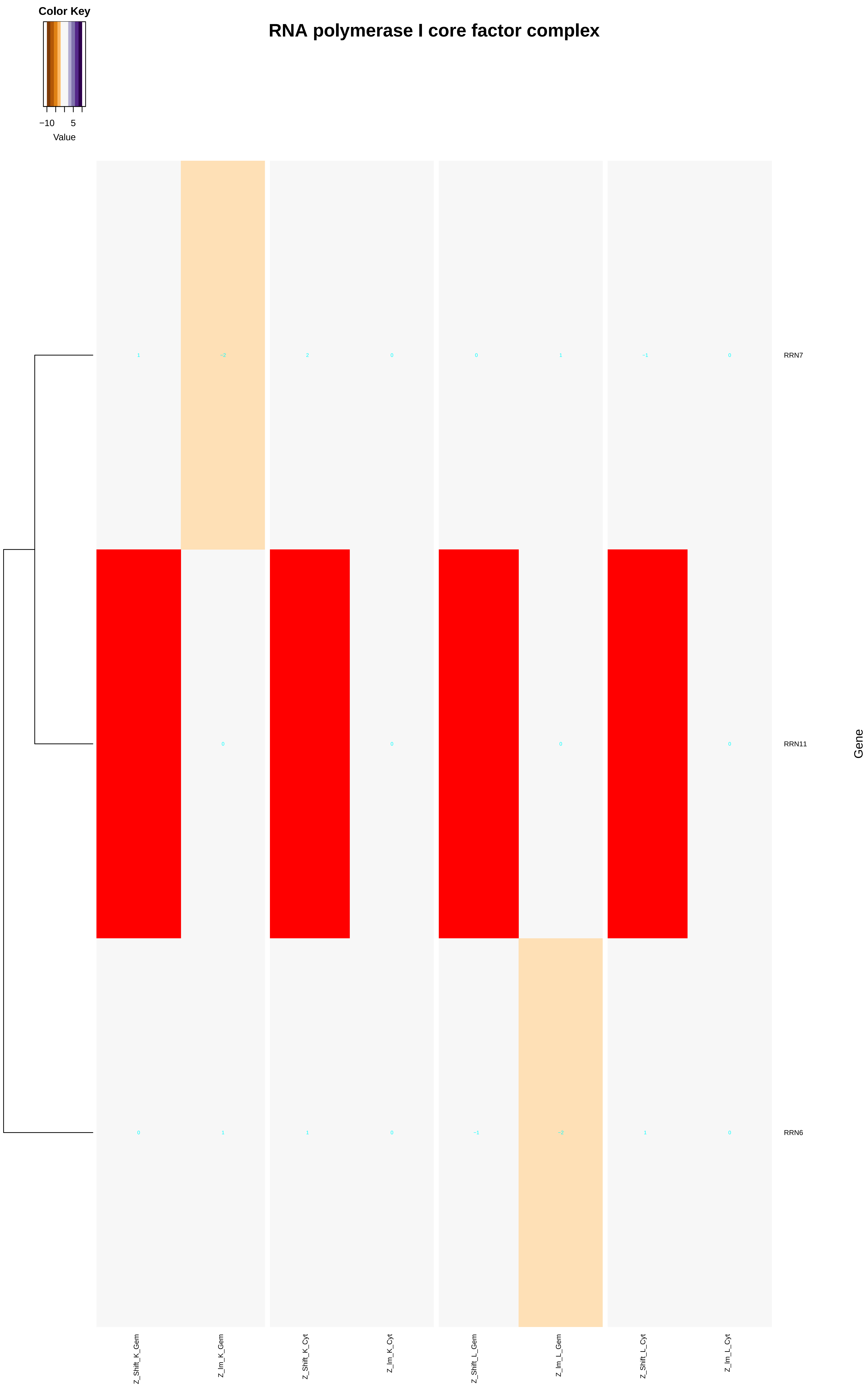
Color Key
−10
5
Value
RNA polymerase I core factor complex
RRN7
RRN11
RRN6
Gene
Z_Shift_K_Gem
Z_Im_K_Gem
Z_Shift_K_Cyt
Z_Im_K_Cyt
Z_Shift_L_Gem
Z_Im_L_Gem
Z_Shift_L_Cyt
Z_Im_L_Cyt

transcription factor TFIID complex

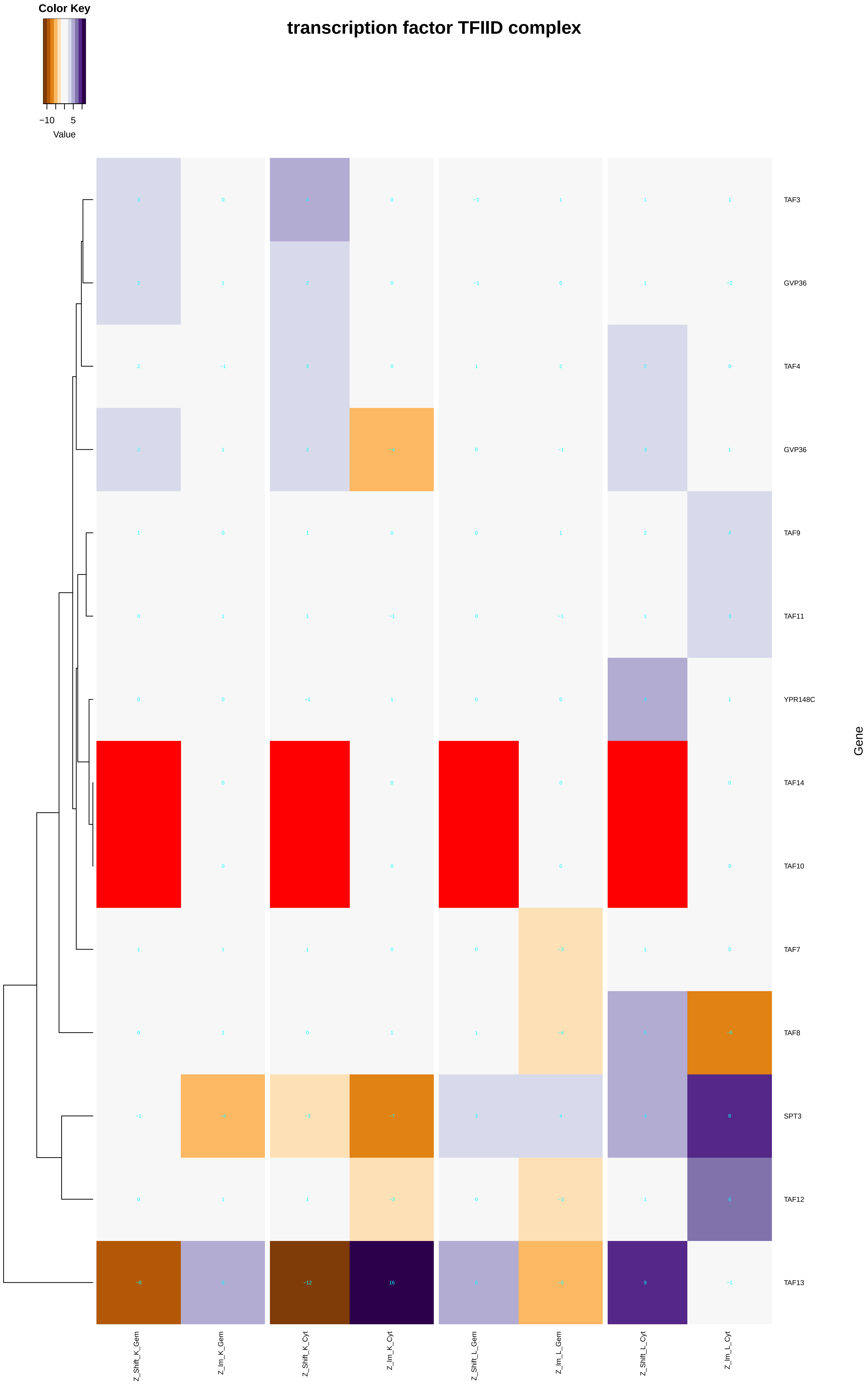

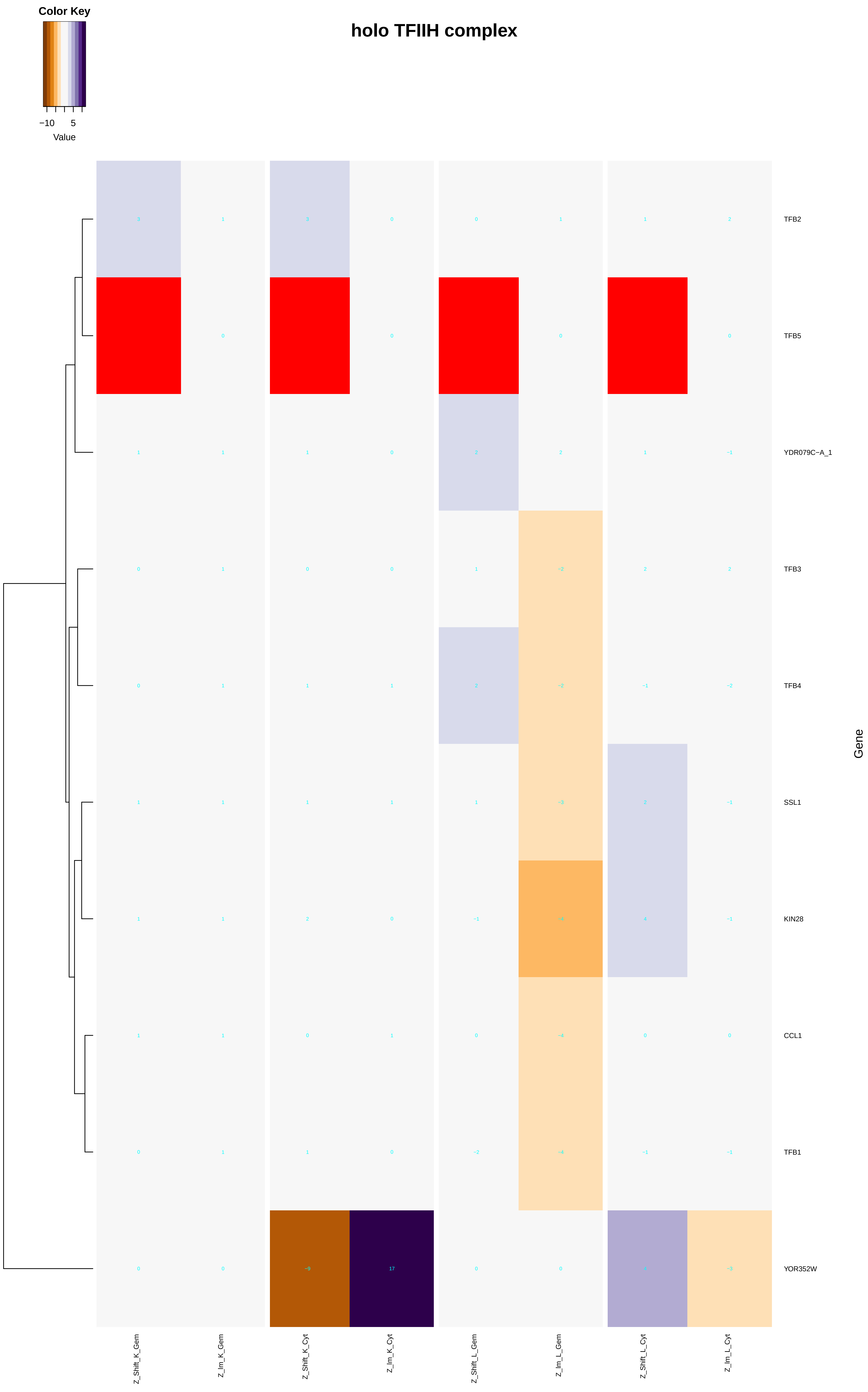

Color Key
−10
5
Value
holo TFIIH complex
TFB2
TFB5
YDR079C−A_1
TFB3
TFB4
SSL1
KIN28
CCL1
TFB1
YOR352W
Gene
Z_Shift_K_Gem
Z_Im_K_Gem
Z_Shift_K_Cyt
Z_Im_K_Cyt
Z_Shift_L_Gem
Z_Im_L_Gem
Z_Shift_L_Cyt
Z_Im_L_Cyt

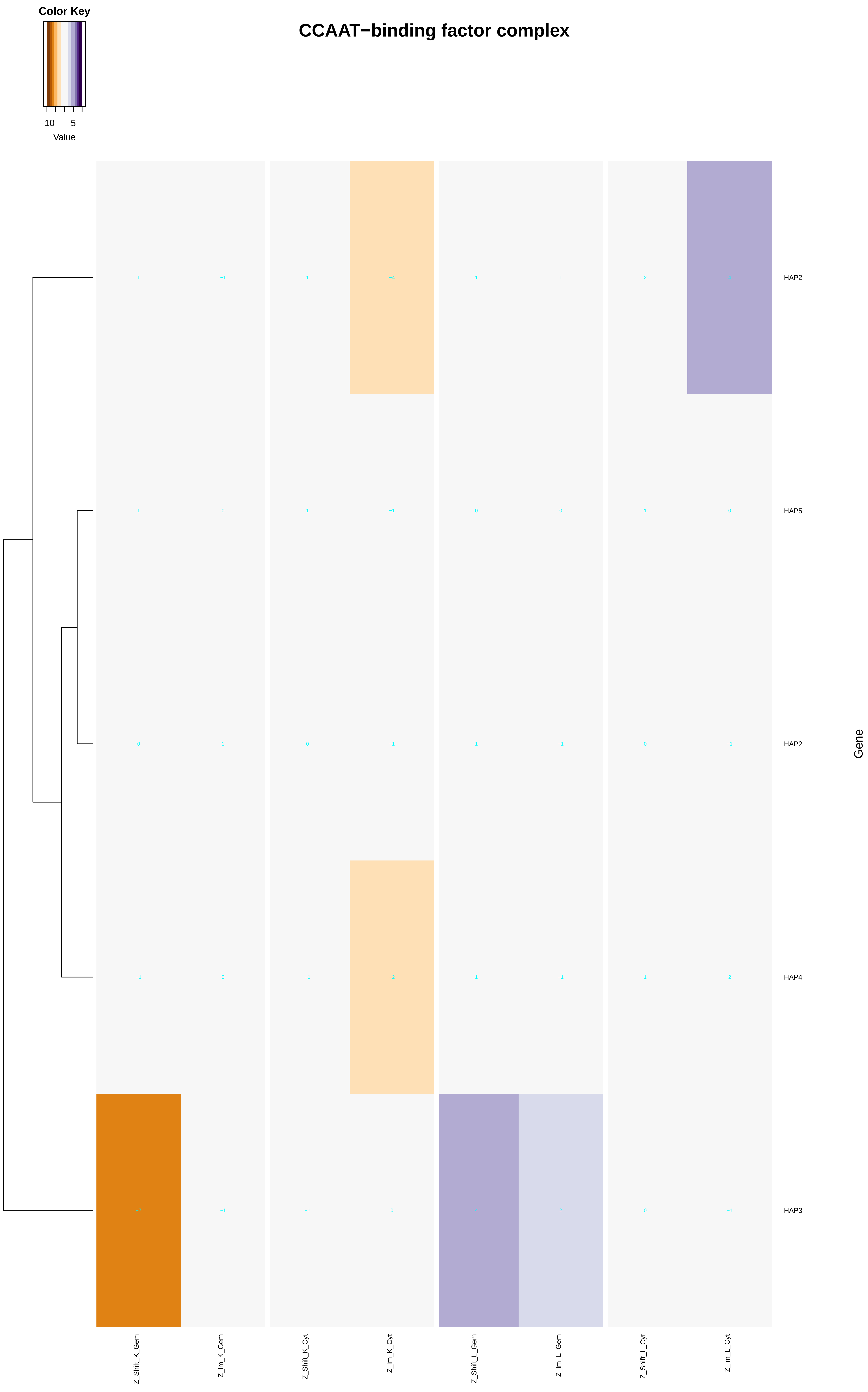

Color Key
−10
5
Value
CCAAT−binding factor complex
HAP2
HAP5
HAP2
HAP4
HAP3
Gene
Z_Shift_K_Gem
Z_Im_K_Gem
Z_Shift_K_Cyt
Z_Im_K_Cyt
Z_Shift_L_Gem
Z_Im_L_Gem
Z_Shift_L_Cyt
Z_Im_L_Cyt

MBF transcription complex

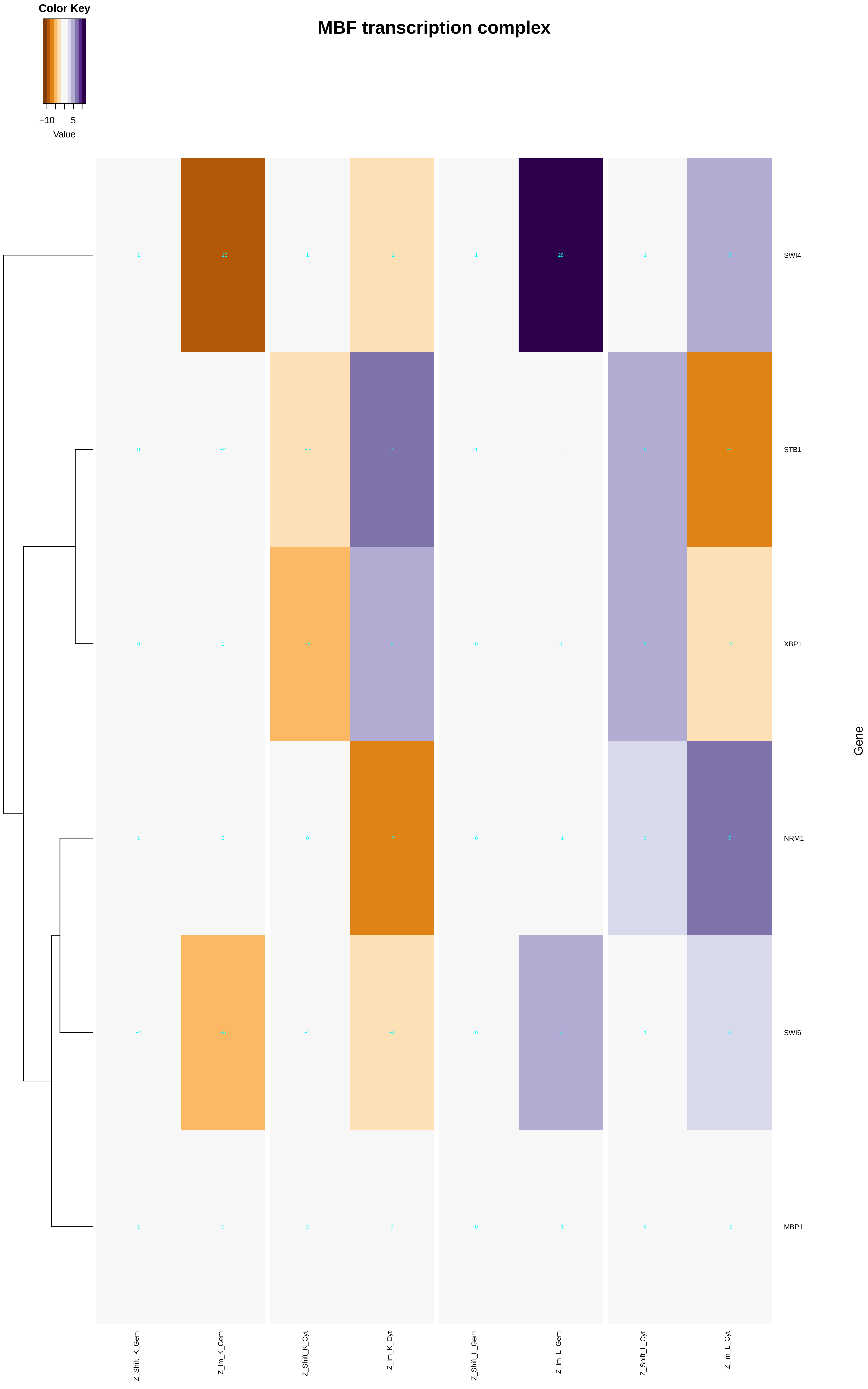

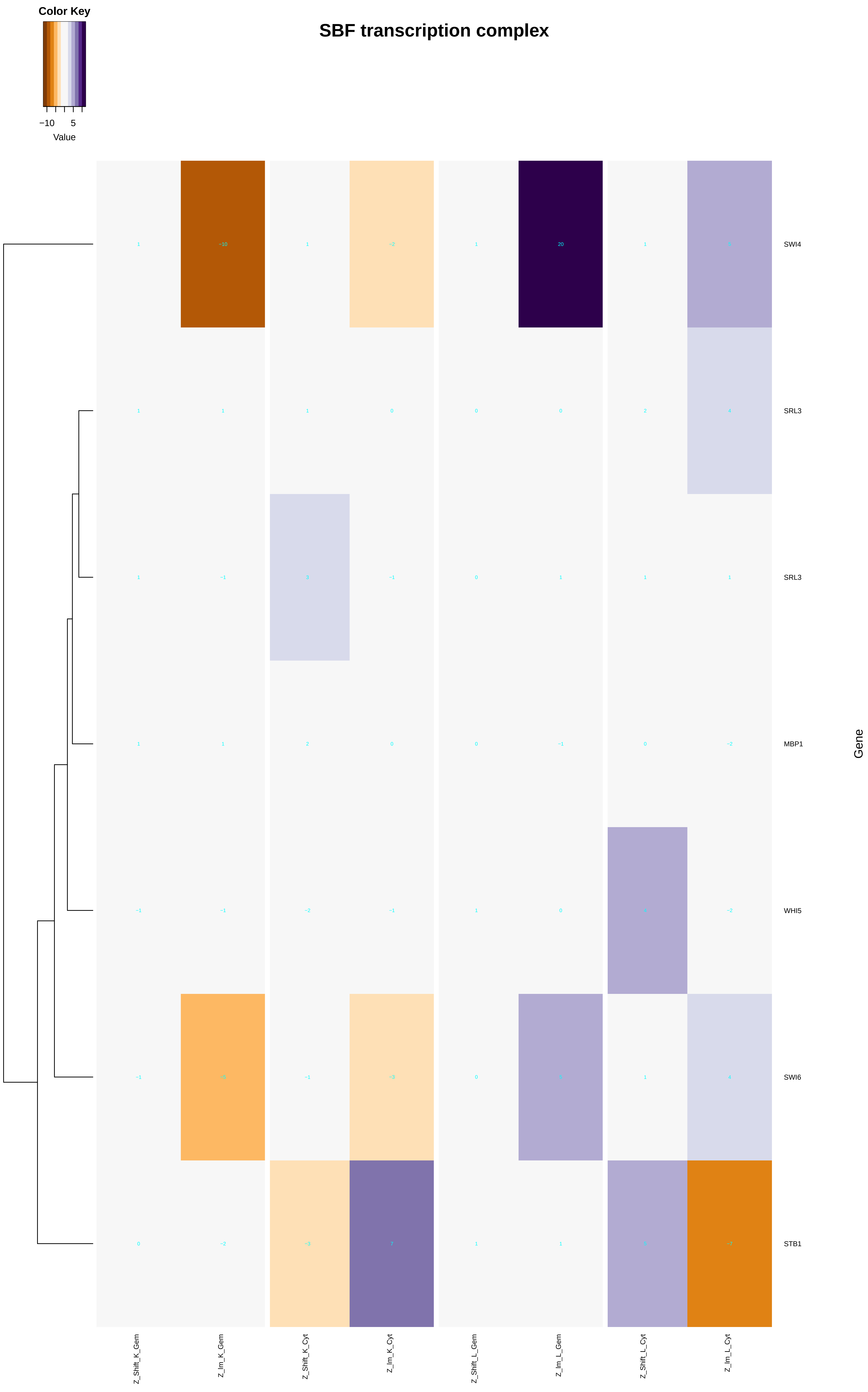
Color Key
−10
5
Value
SBF transcription complex
SWI4
SRL3
SRL3
MBP1
WHI5
SWI6
STB1
Gene
Z_Shift_K_Gem
Z_Im_K_Gem
Z_Shift_K_Cyt
Z_Im_K_Cyt
Z_Shift_L_Gem
Z_Im_L_Gem
Z_Shift_L_Cyt
Z_Im_L_Cyt

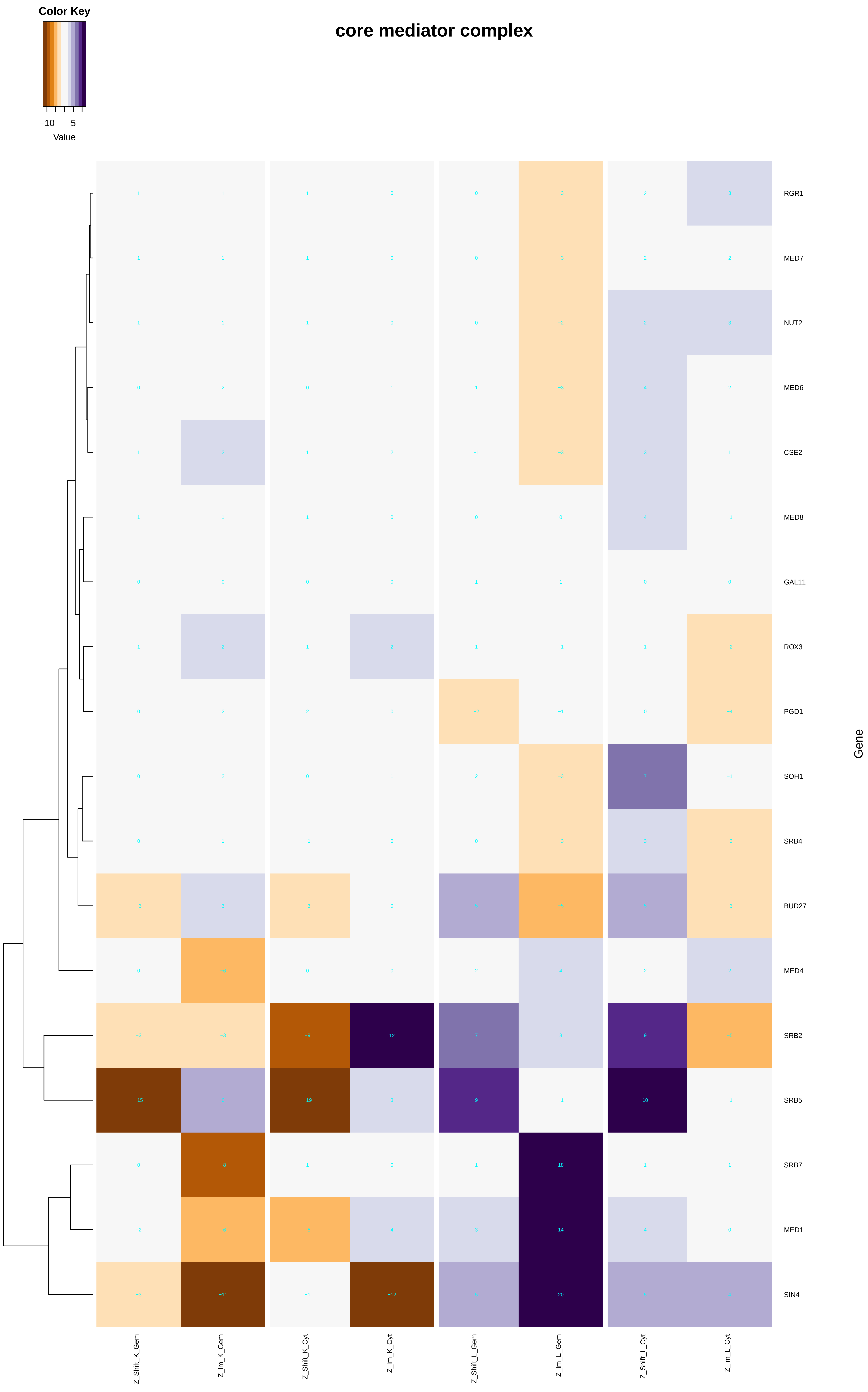
core mediator complex
Color Key
−10
5
Value
RGR1
MED7
NUT2
MED6
CSE2
MED8
GAL11
ROX3
PGD1
SOH1
SRB4
BUD27
MED4
SRB2
SRB5
SRB7
MED1
SIN4
Gene
Z_Shift_K_Gem
Z_Im_K_Gem
Z_Shift_K_Cyt
Z_Im_K_Cyt
Z_Shift_L_Gem
Z_Im_L_Gem
Z_Shift_L_Cyt
Z_Im_L_Cyt

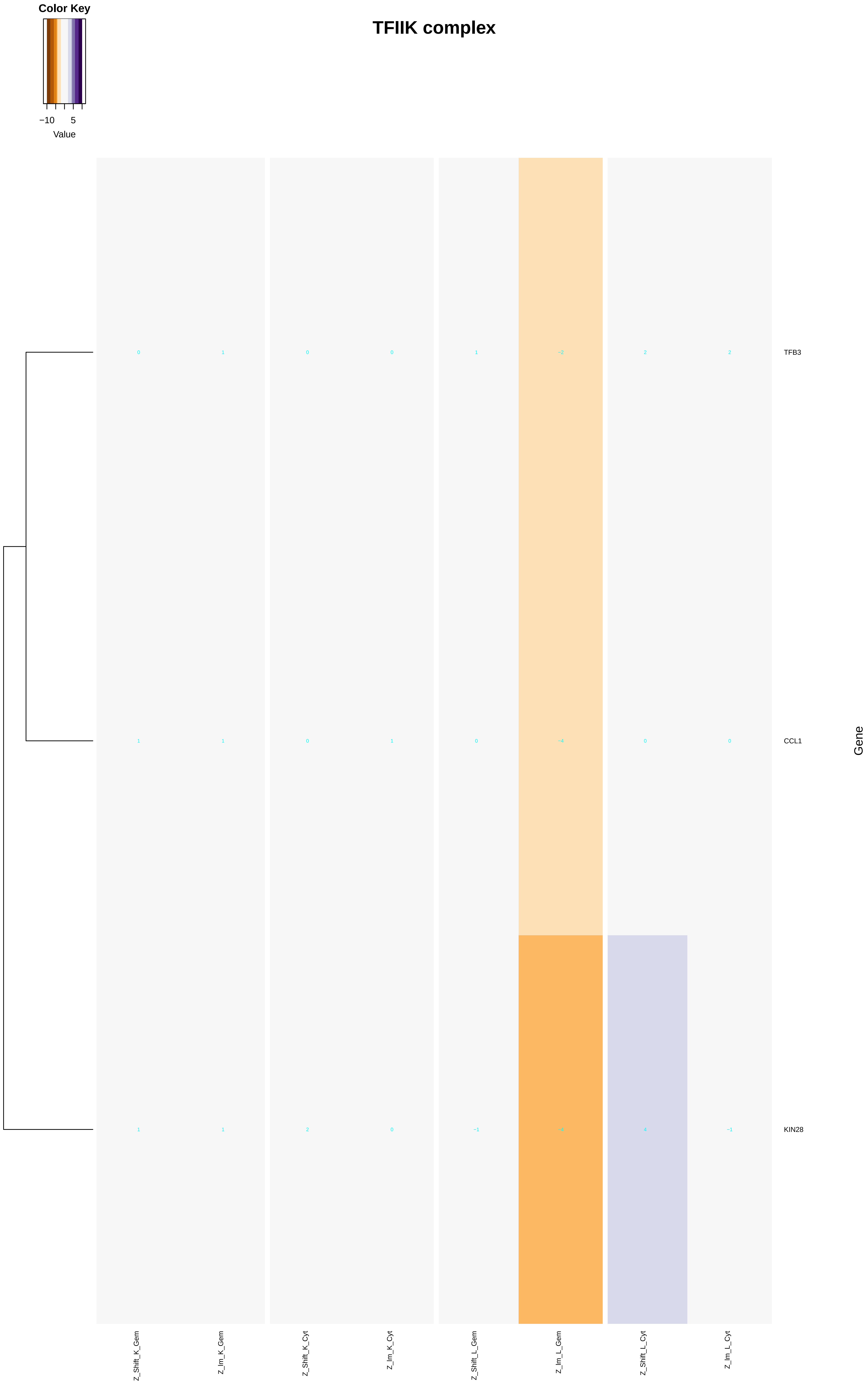
Color Key
−10
5
Value
TFIIK complex
0
1
0
0
1
−2
2
2
TFB3
1
1
0
1
0
−4
0
0
CCL1
1
1
2
0
−1
−4
4
−1
KIN28
Gene
Z_Shift_K_Gem
Z_Im_K_Gem
Z_Shift_K_Cyt
Z_Im_K_Cyt
Z_Shift_L_Gem
Z_Im_L_Gem
Z_Shift_L_Cyt
Z_Im_L_Cyt
